# Worsened outcomes of newly diagnosed cancer in patients with recent emergency care visits: A retrospective cohort study of 3699 adults in a safety net health system

**DOI:** 10.1002/cam4.5303

**Published:** 2022-11-16

**Authors:** Nicholas R. Pettit, Xin Li, Lauren Stewart, Jeffrey Kline

**Affiliations:** ^1^ Department of Emergency Medicine Indiana University Indianapolis Indiana USA; ^2^ Richard M. Fairbanks School of Public Health Indiana University Indianapolis Indiana USA; ^3^ Department of Emergency Medicine Wayne State University Detroit Michigan USA

**Keywords:** accident and emergency medicine, cancer, cancer screening, disparities, oncology

## Abstract

**Introduction:**

Many patients receive a suspected diagnosis of cancer through an emergency department (ED) visit. Time to treatment for a new diagnosis of cancer is directly associated with improved outcomes with little no describing the ED utilization prior to the diagnosis of cancer. We hypothesize that patients that have an ED visit in proximity to a diagnosis of cancer will have worse outcomes, including mortality.

**Methods:**

This study is a retrospective cohort study of all patients with cancer diagnosed at Eskenazi Health (Indiana) between 2016 and 2019. Individual health characteristics, ED utilization, cancer types, and mortality were studied. We compared those patients seen in the ED within 6 months prior to their diagnosis (cases) to patients not seen in the ED (controls).

**Results:**

A total of 3699 patients with cancer were included, with 1239 cases (33.50%). Patients of black race had higher frequencies in the cases vs. controls (46.57% vs. 40.68%). Lung cancer was the most frequently observed cancer among cases vs. controls (11.70% vs. 5.57%). For the cases, 232 patients were deceased (18.72%) compared with 247 patients among the controls (10.04%, *p* < 0.0001, OR 2.06 95% confidence interval [CI] 1.70–2.51). An ED visit in past 6 months (OR = 1.73, 95% CI 1.38–2.18) and Medicaid insurance type (versus commercial, OR = 4.16, 95% CI 2.45–7.07) were associated with of mortality. Female gender (OR = 0.76, 95% CI 0.67–0.88), tobacco use (OR = 1.62, 95% CI 138–1.90), and Medicaid insurance type (versus commercial, OR = 2.56, 95% CI 2.07–3.47) were associated with prior ED use.

**Conclusions:**

Over one third of patients with cancer were seen in the ED within 6 months prior to their cancer diagnosis. Higher mortality rates were observed for those seen in the ED. Future studies are needed to investigate the association and impact that the ED has on eventual cancer diagnoses and outcomes.

## 1 INTRODUCTION

While cancer is a leading cause of death, little is known about the epidemiology and health care utilization prior a diagnosis of cancer, including emergency department (ED) use.^1^ In 2017, approximately 2 million adults nationwide reported the ED as their usual course of health care, with 18 million adults reporting no access to routine medical care.^2^ While cancer is routinely and ideally diagnosed through elective screening or by primary care physicians, 20%–50% of global cancer diagnoses originate from an index ED visit. Higher frequencies of ED‐associated diagnoses are among racial/ethnic minorities and patients of lower socioeconomic status.^3^ Prior work from the United Kingdom's “Routes‐to‐Diagnosis” work suggested that 23% of newly diagnosed cancer patients presented emergently and survival rates were much lower for emergent presenters.^4^ When people present to the ED with life threatening manifestations of their undiagnosed cancer they are more likely advanced stages and are less likely to be curative.^5^, ^6^

Safety‐net hospital systems, including safety‐net EDs, disproportionately serve vulnerable patient populations.^7^ Safety‐net hospitals generally treat greater percentages of racial/ethnic minorities, Medicaid and uninsured patients, patients from communities in the lowest income quartile, and provide large amounts of uncompensated care.^7^ The populations that rely on safety‐net hospital systems for care are the same populations that experience cancer care disparities, including disparities in equitable cancer screening opportunities and less access to the most advanced cancer treatments.^8^ Little descriptive data exists regarding the prevalence of cancer types treated at safety‐net hospitals.^9^ Less is known about the frequency at which patients seek medical care in safety‐net EDs prior to their diagnosis of cancer. We hypothesize that patients diagnosed in proximity to an ED visit have worse outcomes than patients without prior ED use, and these outcomes will be exacerbated by health disparities. This will suggest that the ED visit serves as a proxy for either inadequate health care maintenance/screening, limited access to health care services, or patients will present with more advanced and symptomatic disease.

While increasing evidence and factors associated with emergency presentations of cancer are available^5^, ^10^, ^11^, ^12^, ^13^ (and primarily based out of the United Kingdom [UK]), to our knowledge, there are no administrative‐database studies conducted in the United States (USA) that have examined the characteristics, eventual cancer types, and outcomes of ED utilization during the pre‐diagnosis phase. This context is important, as studies from the UK cannot necessarily be generalized to the USA. This information may help illuminate potential gaps in access, quality, and equity of both cancer screening and the state‐of‐the‐art cancer treatment, especially as they pertain to patients that receive a suspected cancer diagnosis in the ED.

## 2 MATERIALS METHODS

### 2.1 Setting and selection of participants

This was a retrospective study conducted using data from the Eskenazi Health System, the sole safety‐net health system for Indianapolis Indiana. Most patients are low‐income, and most of the patients identify as a racial or ethnic minority. The ED affiliated with the hospital sees greater than 100,000 patients annually, and serves as a level 1 trauma center, major stroke center, and burn hospital for the state of Indiana. All outpatient and inpatient care in the Eskenazi system is captured in its comprehensive electronic health record (EHR, Epic). This study was approved by the Indiana University's Institutional Review Board (IU IRB), #2001716264 prior to commencing the study.

Inclusion criteria included all adult patients 18 years and older in in the EHR at Eskenazi Health, with a first time of a diagnosis of cancer between January 1, 2016 and December 31, 2019. Patients were identified and restricted to the first time a neoplasm/tumor/cancer‐defining ICD code appearing in the EHR within those 3 years. Patients were excluded if the ICD‐related code was for a non‐malignant tumor.

### 2.2 Data collection

Through the use of existing data within the EHR, the following variables were electronically obtained age at time of cancer diagnosis, sex, race, ethnicity, Charlson Comorbidity Index,^14^ the ICD Oncology Topography codes (Table S1), the cancer primary site, mortality, insurance type at time of diagnosis, body mass index (BMI), social history (tobacco use, alcohol use, and illicit drug use), presence of an ED visit, and number of ED visits. Mortality was assessed at 1 year post index ED visit and was all‐cause mortality. The index date for all patients was set as the date of initial cancer diagnosis according to the first posted cancer diagnosis date. Patients seen in the Eskenazi Health ED were flagged by the EHR, and cases were defined as patients with ED visits in the 6 months before their cancer diagnosis were and controls were all others.

### 2.3 Outcome measures

The primary outcome of this study was comparing the percent mortality between cases and controls. Secondary outcomes included the odds of a covariate predicting the likelihood of predicting mortality and an ED visit, as well as the number of ED visits that occurred among the cases. As an exploratory analysis, we sought to identify and characterize patients by cases and controls, and by cancer type.

### 2.4 Statistical analysis

This study is reported as per the Strengthening the Reporting of Observational studies in Epidemiology guidelines.^15^ Patient demographics and clinical characteristics were compared between those patients who did not have an ED visit within 6 months prior to cancer diagnosis (controls) and those patients who did (cases), and were described using frequencies and percentages for categorical data and means with interquartile ranges for continuous variables, as appropriate. To test for differences between groups, the Chi‐square test or Fisher's exact test was used for bivariate variables, and the Wilcoxon test for continuous variables (all at 95% confidence interval). Adjusted odds ratios from logistic regression containing the following dependent variables (age, gender, race, drug use, alcohol use, tobacco use, and the Charlson Comorbidity Index [CCI])^16^ was used to determine the association between having an ED visit before cancer diagnosis and risk of death. When appropriate, we controlled for gender for gender‐specific malignancies. After developing the logistic regression models, sensitivity analysis was performed to assess the robustness of the associations were to potential unmeasured or uncontrolled founding, using the E‐Value.^17^ Additionally, statistical analysis assessing the presence of effect modification was performed, and no interactions between confounders were found to be significant. Thus, no effect modification was observed or reported. The primary outcome was the frequency of patients with cancer seen in the ED. Secondary outcomes include the percent mortality between cases and controls, as well as the resulting odds ratios for variables likely to be associated with both mortality and the likelihood of being seen in the ED prior to a diagnosis of cancer. All statistical analyses were conducted using SAS statistical software. Missing values were not replaced in the data analysis.

## 3 RESULTS

**FIGURE 1:**
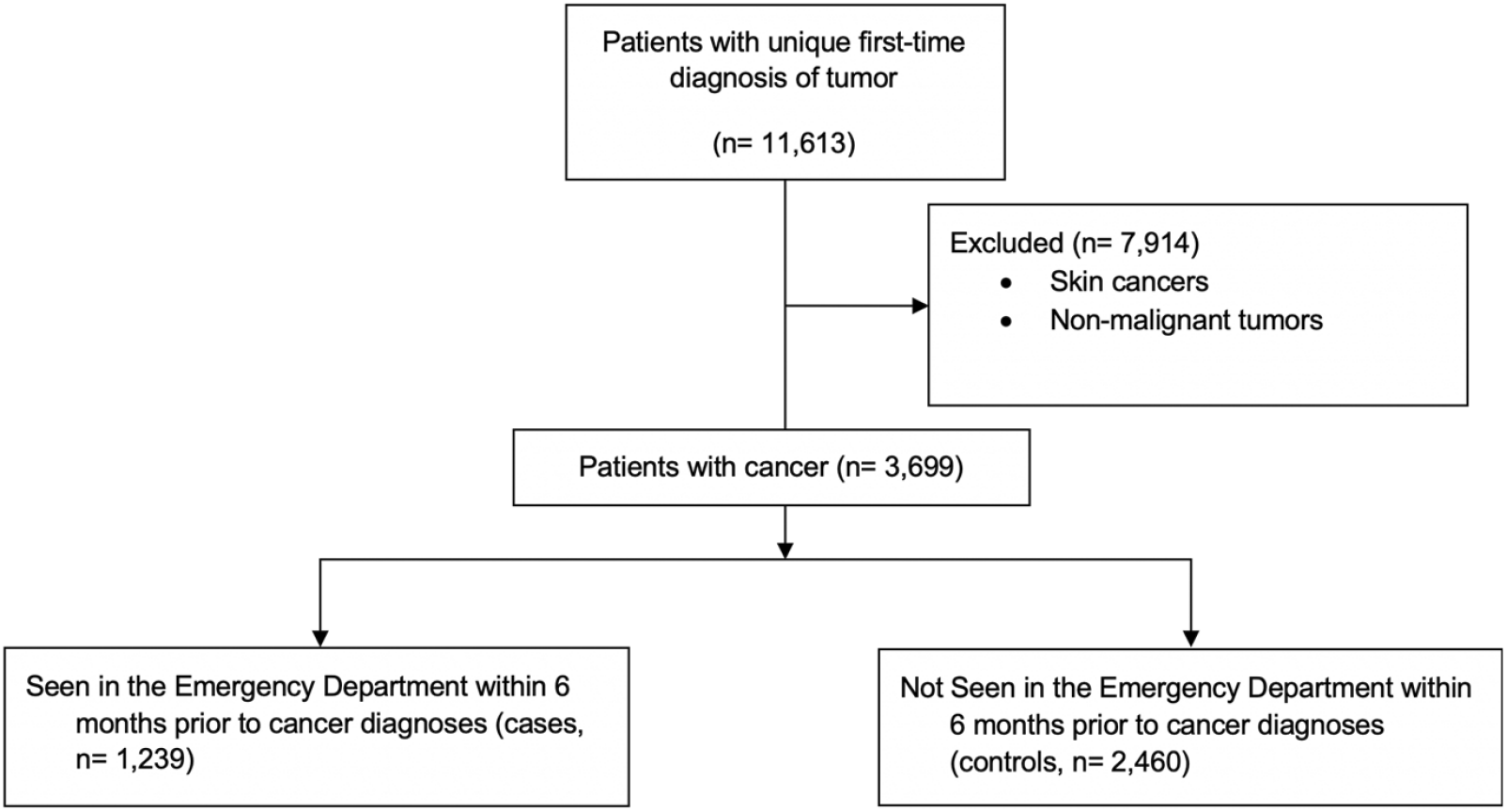
Flowchart describing selection of patients

In total, over the 3‐year study period, 3699 patients with cancer were identified in the EHR, with 1239 (33.50%) cases having had an ED visit within 6 months prior to the diagnosis of cancer. Figure 1 provides a flowchart of the assembly of the study cohort.

### 3.1 Sociodemographic and patient characteristics of the study population

Table 1 presents the demographics and characteristics of the patients involved in this study, separating patients into cases (patients with an associated ED visit within 6 months from the time of cancer diagnosis), and controls (those with no‐associated ED visits in prior 6 months). Pertinent differences between the two groups include controls were more likely to be female (58.70% vs. 52.06%, *p* = 0.0005) with a median age of 58 years old. African Americans comprised a significantly greater proportion of cases when compared with the controls (46.57% vs. 40.68%, *p* = 0.003). The controls had slightly higher rates of ethnically Latinx, compared with controls (15.31% vs. 14.53%, *p* < 0.0001). Furthermore, cases had higher rates of noncommercial insurances, more specifically the Healthy Indiana Plan (low‐cost state offered insurance plan), Medicaid, Medicare, and self‐pay status compared with private/commercial insurance (*p* < 0.0001). Patients who were seen in the ED prior to their diagnoses had higher rates of a family history of lung cancer (4.60% vs. 3.29%). Additionally, patients seen in the ED had significantly higher rates of tobacco use (42.01% vs. 30.90%, *p* = 0.0476) and illicit drug use (11.05% vs. 6.97%, *p* = 0.0005). Lastly, for the cases, the mean number of ED visits prior to a cancer diagnosis was 1.8 (minimum = 1, maximum = 52).

**TABLE 1:** Characteristics of the study population

	Total (*n* = 3699)	Cases (ED visit within 6 months prior) (*n* = 1239, 33.50%)	Controls (no ED visit within 6 months prior) (*n* = 2460, 66.50%)	95% CI for difference
Gender—female	2089 (56.5%)	645 (52.06%)	1444 (58.70%)	3.2%–10.0%
Race				
Black	1568 (42.67%)	577 (46.57)	991 (40.68)	2.7%–9.5%
White	1480 (40.27%)	474 (37.45)	1016 (41.71%)	0.9%–7.6%
Other	627 (17.06%)	198 (15.98)	429 (17.61)	0.9%–4.2%
Ethnicity—Latinx	551 (15.05%)	180 (14.53%)	371 (15.31%)	1.6%–3.2%
Insurance type				
Commercial	581 (15.80%)	110 (8.88%)	471 (19.32%)	8.2%–12.7%
Healthy Indiana plan	613 (16.67%)	240 (19.39%)	373 (15.30%)	1.5%–6.7%
Health advantage	124 (3.37%)	42 (3.39%)	82 (3.36%)	1.2%–1.3%
Incarcerated	18 (0.49)	8 (0.65%)	10 (0.41%)	0.3%–0.8%
Medicaid	699 (19.01%)	269 (21.71%)	430 (17.64%)	1.3%–6.8%
Medicare	1298 (35.27)	453 (36.56%)	844 (34.62%)	2.2%–4.2%
Other	41 (1.12)	9 (0.73%)	32 (1.31%)	0.07%–1.2%
Self‐pay	234 (6.36%)	81 (6.54%)	153 (6.28%)	1.4%–1.9%
Sliding fee	66 (1.79)	25 (2.02%)	41 (1.68%)	0.06%–1.3%
Body Mass Index (BMI, median, IQR)	28.3 (23.6–33.6)	27.7 (22.8–33.1)	28.6 (24.0–33.9)	
Family history				
Lung cancer	138 (3.7%)	57 (4.60%)	81 (3.29%)	0.06%–2.7%
Age at diagnosis (median, IQR)	58 (47.0–65.0)	58 (49.0–65.0)	58 (45.0–65.%)	
Social history^a^				
Tobacco use	1078 (34.9%)	468 (42.01%)	610 (30.90%)	7.8%–14.4%
Alcohol use	709 (26.68%)	239 (25.21%)	470 (27.34%)	0.08%–5.1%
Illicit drug use	216 (8.4%)	100 (11.05%)	116 (6.97%)	2.1%–6.1%
# Previous ED visits, (mean, IQR, max)	1239	1239 (1.8, 1–2, 52)		

Abbreviations: CI, confidence interval; ED, emergency department; IQR, interquartile range; OR, odds ratio.

^a^ Missing values due to additional categories for substance use including “not asked” and “passive”.

### 3.2 Main results

Table 2 lists the top 10 malignancies in the cases cohort in order of decreasing frequency, with Table S2, presenting the entire cohort of cancers. The most common cancer among cases is lung cancer, which is nearly twice as frequently diagnosed than the controls (11.70% vs. 5.57%, *p* < 0.0001). The next four most common cancers diagnosed for the cases were breast cancer (10.17%), head/ear/nose/throat cancers (8.80%), prostate cancer (7.91%), and pancreatic cancer (7.43%), with only pancreatic cancer being more common among the cases versus controls. In total, colorectal, kidney, liver, lung, ocular, pancreatic, and vaginal cancers have statistically higher frequencies of diagnosis among cases than controls. Among cancers that have known screening modalities, breast cancer, prostate cancer are the only two that have statistically higher frequencies among controls than cases when controlling for gender.^18^ Cervical cancer, when controlling for gender, is not statistically different between the cases and controls. Comparatively, colorectal cancer and lung cancer both have effective screening tests, meanwhile patients with these diagnoses have higher observed frequencies among the cases.

**TABLE 2:** Frequency of cancer types observed in this study. Order in decreasing frequency among cases (those with ED visits within 6 months prior to diagnosis)

Cancer type	Total (*n* = 3699)	Cases (ED visit within 6 months prior) (*n =* 1239, 33.5%)	Controls (no ED visit within 6 months prior) (*n* = 2460, 66.5%)	*p* value^a^
Lung	282 (7.62%)	145 (11.70%)	137 (5.57%)	<0.0001
Breast^b^	572 (15.46%)	126 (10.17%, 19.2%))	449 (18.18%, 31.1%)	<0.0001
Head, ears, nose, throat	359 (9.91%)	109 (8.80%)	250 (10.16%)	0.1856
Prostate^b^	312 (8.43%)	98 (7.91%, 16.5%)	214 (8.70%, 21.1%)	0.4147 (0.025)
Pancreas	200 (5.41%)	92 (7.43%)	108 (4.39%)	0.0001
Colorectal	209 (5.65%)	85 (6.86%)	124 (5.04%)	0.0237
Cervical^b^	235 (6.33%)	63 (5.08%, 9.60%)	171 (6.95%, 11.84%)	0.028 (0.13)
Metastatic unknown primary	180 (4.87%)	57 (4.60%)	123 (5.0%)	0.594
Liver	103 (2.78%)	54 (4.28%)	50 (2.03%)	<0.0001
Kidney	123 (3.33%)	53 (4.28%)	70 (2.85%)	0.0218

Abbreviation: ED, emergency department.

^a^ Estimated by chi‐square test. Column values are the total n in that cohort per cancer type, followed by the column frequency.

^b^ For cancer types with an additional value in paratheses, we compared cases with controls controlling for gender.

The primary outcome of this study is comparing the mortality among patients seen in the ED prior to diagnosis compared with those not seen in the ED. The top half of Table 3 presents the unadjusted and adjusted odds ratios for the prediction of mortality between cases and controls performing logistic regression. For the cases, 232 patients were deceased (18.72%) compared with 247 patients (10.04%) among the controls (*p* < 0.0001, OR 2.06 95% CI 1.70–2.51). Modeling the outcome mortality against gender, age, ED visit, CCI, insurance type, ethnicity, race, tobacco use, and alcohol use, only race and tobacco/alcohol use were not significant predictors and subsequently removed from the model. Table 3 demonstrates the unadjusted and adjusted OR and 95% confidence intervals demonstrating age, ED visit, CCI, insurance type predicting mortality. Secondarily, in the bottom half of Table 3, a secondary outcome is the ability to predict an ED visit. Among the same mentioned variables above, gender, CCI, insurance type, and tobacco use are significant predictors of an ED visit, with insurance type yielding the largest odds ratio of a being significant predictor of an ED visit.

**TABLE 3:** Logistic regression analysis. Top frame is predicting mortality and bottom frame is predicting ED the odds of being a case or having an ED visit

Predicting mortality			
	Unadjusted OR (95% CI)	Adjusted OR (95% CI)*	E‐Value (CI)
Gender (female vs. male)	0.57 (0.47–0.69)	0.70 (0.56–0.88)	2.13 (1.53)
Age	1.04 (1.03–1.05)	1.03 (1.02–1.04)	1.15 (1.16)
ED visit in past 6 months (yes vs no)	2.06 (1.70–2.51)	1.73 (1.38–2.18)	2.27 (2.10)
Charlson comorbidity Index (yes vs. no)	1.12 (1.08–156)	1.05 (1.01–1.10)	1.21 (1.11)
Insurance type (reference = commercial/private)			
Healthy Indiana plan	2.10 (1.30–340)	2.02 (1.13–3.61)	2.79 (1.51)
Medicaid	4.24 (2.85–6.87)	4.16 (2.45–7.07)	5.62 (4.33)
Medicare	4.50 (2.95–6.85)	2.54 (1.50–4.30)	3.51 (2.37)
Self‐pay	3.14 (1.81–5.44)	3.52 (1.83–6.67)	4.80 (3.06)

Predicting ED visit			
	Unadjusted OR (95% CI)	Adjusted OR (95% CI)	
Gender (female vs. male)	0.76 (0.67–0.88)	0.84 (0.71–0.99)	1.51 (1.11)
Charlson Comorbidity Index (yes vs. no)	1.09 (1.07–123)	1.08 (1.05–1.12)	1.28 (1.28)
Insurance type(reference = commercial/private)			
Healthy Indiana plan	2.76 (2.12–3.59)	2.51 (1.84–3.43)	3.47 (3.08)
Medicaid	2.68 (2.07–3.47)	2.48 (1.83–3.36)	3.43 (3.06)
Medicare	2.30 (1.81–291)	1.98 (1.48–2.63)	2.74 (2.32)
Self‐Pay	2.27 (1.61–3.19)	2.55 (1.69–3.82)	3.53 (2.77)
Tobacco use (yes vs. never)	1.62 (1.38–190)	1.39 (1.15–1.66)	1.85 (1.57)

Abbreviations: CI, confidence interval; ED, emergency department; OR, odds ratio.

^a^ Initial model included gender, age, ED visit in past 6 months (mortality predictor only), Charlson Comorbidity Index, insurance type, and tobacco use. Non‐significant predictors removed from model.

## 4 DISCUSSION

Patients who present to the ED with life threatening manifestations of their undiagnosed cancer are known to present with advanced stage malignancies and ultimately suffer higher mortality rates than those diagnosed through non‐emergent pathways.^5^ Most literature in the space of emergency presentations of undiagnosed cancer is based out of the United Kingdom.^5^ In this work we begin to investigate the association between ED visits and a subsequent diagnosis of cancer by describing and comparing those patients with an ED visit prior to the diagnosis of cancer to those that have no ED visits within our safety‐net health network. We demonstrate gender, age, insurance, social history (tobacco use, alcohol use) differences among those that use the ED and are subsequently diagnosed with cancer. Additionally, we demonstrate the most common cancers collectively seen at our safety‐net hospital, with significant differences among cases and controls for many screening‐preventable malignancies. Little to no literature exists examining the association with eventual cancer diagnosis in association with an ED visit, offering opportunities for addressing cancer prevention and cancer screening in the context of an ED visit.^19^

Describing post‐cancer diagnosis ED utilization has been studied, and anywhere from 2% to 4% of all ED visits are attributable toward persons with a diagnosis of cancer.^20^ As cancer survival rates increase, the number of cancer‐related ED visits also increases, and future work is needed to improve cancer management in the ED and potentially reduce unnecessary ED visits.^20^, ^21^ Prior ED use among patients with cancer is the strongest independent predictor for future ED use after a new diagnosis.^22^ Since ICD‐coded cancer diagnoses require tissue sample and biopsy, little to no confirmed cancer diagnoses originate from an ED visit. Instead, many patients in the ED are discharged with symptom‐based discharge diagnoses, and thus linking a subsequent cancer diagnosis to an exact ED‐visit is challenging in administrative databases.^23^ Thus, our work adds to the current literature by describing the population (in a safety‐net setting) that is diagnosed with cancer, and what major cancer types are diagnosed shortly after an ED visit.

Lung, pancreatic, colorectal, liver, and kidney cancers are all seen at higher rates in the ED prior to the diagnosis of cancer, suggesting that some undiscovered moderators may be integral to this problem, such as tobacco use among underserved populations.^24^ Tobacco use is linked to many cancers and in this work there is a higher observed frequency of tobacco use among cases compared with controls (42.0% vs. 30.9%, *p* < 0.0001).^25^ EDs frequently provide care to populations that use higher tobacco at higher rates than the general public.^26^ Thus, it is not surprising that the most common cancers seen in our cases cohort are those that are heavily mediated by tobacco use, namely lung, HENT, pancreatic, colorectal, liver, and kidney. These results are similar compared with a statewide epidemiologic assessment we previously performed, again demonstrating that tobacco‐related cancers appear to be associated with ED visits.^10^, ^24^ Randomized clinical trials have demonstrated success at improving tobacco abstinence in low‐income ED smokers, and our work demonstrates further need for wide spread tobacco cessation programs and cancer prevention education programs which could possibly be facilitated out of the ED.^19^, ^27^ Additionally, this work provides for the data needed to selectively target those cancers that are more likely to be suspected in an ED visit, namely lung, breast, prostate, colorectal, and cervical cancers due to their prevalence of being seen in the ED prior to their diagnosis.

The presence of an ED visit prior to a cancer diagnosis in this work does not necessarily represent causation of an eventual diagnosis, but instead confers an opportunity for an ED physician to help transition the care of a patient with likely cancer. For example, lung cancer was the most common cancer seen in the ED prior to an eventual diagnosis, and whether that ED visit was associated with the diagnosis is unclear. One year survival rates for lung cancer can be as low as 15% when stage IV (compared to 85% stage I), and earlier detection is known to be associated with improved survival rates.^28^ Delays in cancer treatment are common, and even a 4‐week delay of cancer treatment is associated with increased mortality across surgical, systemic treatment, and radiotherapy indications for seven cancers.^29^ Patients with lung cancer can experience delays upwards of 4–5 months from first diagnostic test until a definitive diagnosis.^30^ It remains unclear how the involvement of the ED physician, ED‐associated diagnostic test, and transitions of care from the ED visit are associated with these delays. It stands to reason that many ED patients observe delays in their care that could be prevented by improving and addressing the transitions of care for a newly suspected cancer diagnosis.

Cancer screening exists for many of the most prevalent cancer types, such as breast, colorectal, lung, cervical, and prostate cancers, with other focused screenings for various genetic‐based cancers.^31^ The primary goal of cancer screening is to detect a malignancy before it is large enough to result in symptoms, which usually occur at more advanced stages. Breast cancer screening has a high rate of screening attendance in the United States, with over 76% of females having reported a mammogram within the last 2 years.^32^ This likely explains why a higher percentage of the controls are diagnosed with breast cancer than are cases. The uptake of lung cancer screening with yearly low‐dose computed tomography (LDCT) is much lower than observed for breast cancer, with only <6% of screening‐eligible smokers being screened.^33^ Recent guideline changes in lung cancer screening eligibility were made in an attempt to ameliorate disparities in lung cancer screening access for many populations. It is hypothesized that the lung cancer screening attendance rate will decrease due to the large number of newly expected lung cancer screening eligible Americans.^34^ Herein, a large number of patients with subsequently diagnosed lung cancer, and with ongoing tobacco use, utilize the ED in some capacity. Nationally, millions of Americans in need of recommended cancer screening are cared for in EDs, many of whom are hard to reach through other healthcare settings.^19^ Thus, the ED may potentially serve as a novel location to help addresss disparities in cancer screening uptake by improving access to cancer screening and prevention.^19^

Compared with private insurance, patients with Medicaid have been shown to present with more advanced cancer at time of diagnosis and are less likely to receive curative therapies, such as for prostate cancer.^35^ ED usage is high among uninsured and Medicaid patients.^36^ Lack of insurance is associated with lower rates of preventive care, delays in necessary care, and overall mortality in many studies.^36^, ^37^ Thus, it is not unexpected that insurance status, when compared with commercial insurance as a usual payer source, is associated with both mortality and the odds of being seen in the ED within 6 months of diagnosis. Patients with non‐private insurance have been previously shown to be more likely diagnosed with cancer at advanced stages and have worse survival, possibly due to not receiving appropriate and timely cancer screening, diagnosis, and quality care.^38^

Many variables were determined to be confounders in both predicting mortality and the likelihood of having an ED visit prior to the diagnosis of cancer. None of the studied variables had statistically significant associations with each other, and thus no effect modification was observed. However, many unobserved confounders were not included in this study, such as access to prior health care, access to prior cancer screening, socioeconomic status, access to transportation, education, and metabolic syndrome. Many of these unobserved variables are likely influential in the diagnosis of cancer through an ED visit, such as access to prior health care use. Medicaid patients have greater challenges in healthcare access compared with patients with private insurance, and thus knowing many of the patients in this study have Medicaid insurance it is likely that many patients do not have reliable primary care.^39^ Access to equitable cancer screening opportunities varies depending on race, socioeconomic status, and other disparities, and many underserved populations do not have equitable access to cancer screening.^40^ Eskenazi Health serves a primarily underserved population as the main safety‐net hospital system for the greater Indianapolis metropolitan area. In our results, many are not generalizable toward all EDs, however, our population represents the poorest areas of Indiana (both rural and urban Indiana), and thus likely are representative of many rural and urban EDs across the United States.

Various types of cancers are more commonly associated with ED visits than others, along with certain risk factors such as insurance status, tobacco use, male gender, and overall CCI being more likely to be a case than a control. Many of the cancers in this cohort do not have a recognized screening modalities, likely due to their rare occurrences, such as penis cancer. Thus, future work will begin to focus on the more common ED‐associated cancers, such as lung and colorectal. Lung and colorectal cancers do not observe as high rate of screening attendances as breast, and thus future efforts are needed to expand access and awareness for these cancers screenings.^41^ Exploring prior heath care utilization by patients with cancer will offer opportunities to improve cancer care delivery in the ED.

## 5 LIMITATIONS

Several limitations exist in this study, including the retrospective nature by which the data were obtained. This method of data collection does not account for cancer stage at diagnosis, or if the prior ED visit was related to the eventual cancer diagnosis. Further, many patients present to the ED with undiagnosed cancer and are subsequently discharged from the ED with outpatient follow‐up.^24^ We chose 6 months as the time cutoff to be a case, as many patients experience significant delays (months) in cancer staging and treatment initiation. In our work, 33.5% of patients with cancer in our system were seen in our ED prior to their eventual diagnosis of cancer, which is in accordance with reported frequencies by which cancer diagnoses present emergently.^3^ Thus, it is likely that some, if not many (especially for lung cancer), cancer diagnoses originate from an ED visit, and we attempted to capture any and all cases by extending our case definition to 6 months. Additionally, we relied on the ICD‐10 codes for obtaining our cohort, which may lead to bias in our sample selection. Patients with cancer frequently visit the ED with end‐of‐life presentations, many of whom go undiagnosed and never receive biopsy‐confirmed cancer.^42^ The burden of end‐of‐life manifestations of undiagnosed cancer that present acutely to the ED is unknown; however, would likely worsen the outcomes in this study if we could include them in our cases cohort.

We did not account for previous cancer screenings and the impact that those screenings may have on the ultimate diagnosis. Many of the cancer types have multiple methods and opportunities for screening. For example, to screen for colorectal cancer patients can undergo a colonoscopy, flexible sigmoidoscopy, or even fecal occult blood testing. Lastly, due to the heterogeneity of cancer types studied, we did not analyze the treatment types (surgical, immunotherapy, chemotherapy) and its impact on survival, although we surmise that ED‐associated cancer diagnoses would receive fewer curative therapies.

## 6 CONCLUSIONS

Patients with an eventual cancer diagnosis, in our safety‐net health care setting, frequently utilize the ED prior to their diagnosis. Those who utilize the ED prior to their diagnosis of cancer are more likely to be black, are more likely to not have commercial insurance, and have worsened outcomes than those who do not use the ED. Among those who are seen in the ED prior to an eventual diagnosis, lung cancer is overwhelmingly the most common cancer seen in the ED. Lastly, having an ED visit prior to the diagnosis of cancer is likely a proxy for either inadequate health care maintenance/screening, limited access to health care services, and is associated with more advanced disease at time of diagnosis. Future work should explore the disparities observed among those who utilize the ED prior to the diagnosis of cancer, and interventions will be designed to help reduce the cancer care disparities observed in the ED. In conclusion, many people utilize the ED prior to a biopsy‐confirmed cancer diagnosis, offering an opportunity to address cancer prevention, cancer diagnosis, and cancer screening in the ED setting.

## AUTHOR CONTRIBUTIONS

Nicholas R Pettit conceived the study, designed the study, and obtained IRB approval. Nicholas R Pettit, Xin Li, Jeffrey Kline managed the data, assisted in drafting of the manuscript, and contributed to the revision. Nicholas R Pettit performed statistical analysis.

## FUNDING INFORMATION

None.

## CONFLICT OF INTEREST

The authors have no disclosures.

## ETHICS APPROVAL

This study was approved by the Indiana University's Institutional Review Board (IU IRB), #2001716264 prior to commencing the study.

## LAY SUMMARY

The emergency department serves as a common health care setting where patients with an eventual diagnosis of cancer are seen prior to their diagnosis. The association between an emergency department visit and the eventual diagnosis of cancer in terms of cancer type and mortality is unknown. In this retrospective cohort study involving 3699 patients with non‐skin cancers at an urban safety‐net hospital, 33.50% are seen in the emergency department prior to their diagnosis of cancer, with lung cancer being the most common cancer type seen in the emergency department. The emergency department visit is an independent risk factor for mortality.

## PRECIS FOR USE IN THE TABLE OF CONTENTS

Over one third of patients with cancer (1239/3699) were seen in the ED within 6 months prior to their cancer diagnosis. Higher mortality rates were observed for those seen in the ED and those without commercial insurance.

## Supporting information


Table S1–S2

## Data Availability

The data that support the findings of this study are available from the corresponding author upon reasonable request.
