# Supplementary material for: Worsened outcomes of newly diagnosed cancer in patients with recent emergency care visits: A retrospective cohort study of 3699 adults in a safety net health system
Source: Cancer Med. 2022 Nov 16;12(4):4832–41. doi: 10.1002/cam4.5303 (PMC9972123; doi:10.1002/cam4.5303)
Supplement: Supplementary file 1 — Table S1–S2 [file CAM4-12-4832-s001.docx]

| **Supplementary Table 1.** List of ICD codes and cancer type included in this study | |
| --- | --- |
| C01 | Cancer of base of tongue (CMS/HCC) |
| C02.1 | Cancer of tip and lateral border of tongue (CMS/HCC) |
| C02.3 | Malignant neoplasm of anterior two-thirds of tongue (CMS/HCC) |
| C02.9 | Tongue cancer (CMS/HCC) |
| C04.0 | Cancer of anterior portion of floor of mouth (CMS/HCC) |
| C04.9 | SCC (squamous cell carcinoma of floor of mouth) (CMS/HCC) |
| C05.1 | Squamous cell carcinoma of soft palate (CMS/HCC) |
| C06.0 | Squamous cell cancer of buccal mucosa (CMS/HCC) |
| C06.1 | Malignant neoplasm of vestibule of mouth (CMS/HCC) |
| C06.9 | Primary oral squamous cell carcinoma (CMS/HCC) |
| C07 | Cancer of parotid gland (CMS/HCC) |
| C08.0 | Mucoepidermoid carcinoma of submandibular gland (CMS/HCC) |
| C08.9 | Adenocarcinoma of salivary gland (CMS/HCC) |
| C09.9 | Squamous cell carcinoma of right tonsil (CMS/HCC) |
| C10.9 | Oropharyngeal carcinoma (CMS/HCC) |
| C11.9 | Nasopharyngeal carcinoma (CMS/HCC) |
| C13.9 | Malignant neoplasm of hypopharynx (CMS/HCC) |
| C14.0 | Throat cancer (CMS/HCC) |
| C15.3 | Malignant neoplasm of upper third of esophagus (CMS/HCC) |
| C15.5 | Malignant neoplasm of lower third of esophagus (CMS/HCC) |
| C15.9 | Esophageal adenocarcinoma (CMS/HCC) |
| C16.0 | Squamous cell cancer of gastroesophageal junction (CMS/HCC) |
| C16.2 | Malignant neoplasm of body of stomach (CMS/HCC) |
| C16.3 | Malignant neoplasm of pyloric antrum (CMS/HCC) |
| C16.8 | Malignant neoplasm of overlapping sites of stomach (CMS/HCC) |
| C16.9 | Malignant neoplasm of stomach, unspecified location (CMS/HCC) |
| C17.0 | Duodenal adenocarcinoma (CMS/HCC) |
| C17.9 | Goblet cell carcinoid of small intestine (CMS/HCC) |
| C18.0 | Adenocarcinoma of cecum (CMS/HCC) |
| C18.1 | Malignant neoplasm of appendix (CMS/HCC) |
| C18.2 | Malignant neoplasm of ascending colon (CMS/HCC) |
| C18.3 | Malignant neoplasm of hepatic flexure (CMS/HCC) |
| C18.4 | Malignant neoplasm of transverse colon (CMS/HCC) |
| C18.5 | Malignant neoplasm of splenic flexure (CMS/HCC) |
| C18.6 | Malignant neoplasm of descending colon (CMS/HCC) |
| C18.7 | Malignant neoplasm of sigmoid colon (CMS/HCC) |
| C18.8 | Overlapping malignant neoplasm of colon (CMS/HCC) |
| C18.9 | Adenocarcinoma of colon (CMS/HCC) |
| C18.9, C77.2 | Colon cancer metastasized to intra-abdominal lymph node (CMS/HCC) |
| C18.9, C78.7 | Metastatic colon cancer to liver (CMS/HCC) |
| C18.9, C79.9 | Neoplasm of colon, distant metastasis staging category M1a: metastasis confined to one organ or site (CMS/HCC) |
| C19 | Colorectal cancer (CMS/HCC) |
| C20 | Rectal adenocarcinoma (CMS/HCC) |
| C20, C77.5 | Rectal cancer metastasized to intrapelvic lymph node (CMS/HCC) |
| C20, C78.7 | Rectal cancer metastasized to liver (CMS/HCC) |
| C21.0 | Anal cancer (CMS/HCC) |
| C22.0 | Hepatocellular carcinoma (CMS/HCC) |
| C22.1 | Cholangiocarcinoma (CMS/HCC) |
| C22.2 | HBL (hepatoblastoma) (CMS/HCC) |
| C22.7 | Other specified carcinomas of liver (CMS/HCC) |
| C22.8 | Cancer, liver, primary (CMS/HCC) |
| C22.9 | Malignant neoplasm of liver, unspecified liver malignancy type (CMS/HCC) |
| C23 | Gallbladder cancer (CMS/HCC) |
| C24.0 | Klatskin's tumor (CMS/HCC) |
| C24.1 | Ampullary carcinoma (CMS/HCC) |
| C25.0 | Malignant neoplasm of head of pancreas (CMS/HCC) |
| C25.1 | Malignant neoplasm of body of pancreas (CMS/HCC) |
| C25.2 | Malignant neoplasm of tail of pancreas (CMS/HCC) |
| C25.3 | Malignant neoplasm of pancreatic duct (CMS/HCC) |
| C25.4 | Cancer of the endocrine pancreas (CMS/HCC) |
| C25.8 | Overlapping malignant neoplasm of pancreas (CMS/HCC) |
| C25.9 | Malignant neoplasm of pancreas, unspecified location of malignancy (CMS/HCC) |
| C25.9, C78.7 | Pancreatic carcinoma metastatic to liver (CMS/HCC) |
| C26.9 | GI malignancy (CMS/HCC) |
| C30.0 | Primary squamous cell carcinoma of nasal cavity (CMS/HCC) |
| C30.0, C30.1 | Malignant neoplasm of nasal cavity and middle ear (CMS/HCC) |
| C31.1 | Carcinoma of ethmoid sinus (CMS/HCC) |
| C31.2 | Frontal sinus carcinoma (CMS/HCC) |
| C32.0 | Squamous cell carcinoma of right vocal cord (CMS/HCC) |
| C32.1 | Primary cancer of supraglottis (CMS/HCC) |
| C32.8 | Malignant neoplasm overlapping larynx site (CMS/HCC) |
| C32.9 | Squamous cell carcinoma of larynx (CMS/HCC) |
| C34.00 | Malignant neoplasm of hilus of lung, unspecified laterality (CMS/HCC) |
| C34.01 | Malignant neoplasm of hilus of right lung (CMS/HCC) |
| C34.02 | Malignant neoplasm of hilus of left lung (CMS/HCC) |
| C34.10 | Malignant neoplasm of upper lobe, unspecified bronchus or lung (CMS/HCC) |
| C34.11 | Malignant neoplasm of upper lobe of right lung (CMS/HCC) |
| C34.12 | Malignant neoplasm of upper lobe of left lung (CMS/HCC) |
| C34.2 | Malignant neoplasm of middle lobe of right lung (CMS/HCC) |
| C34.30 | Malignant neoplasm of lower lobe of lung, unspecified laterality (CMS/HCC) |
| C34.31 | Malignant neoplasm of lower lobe of right lung (CMS/HCC) |
| C34.32 | Malignant neoplasm of lower lobe of left lung (CMS/HCC) |
| C34.80 | Malignant neoplasm of overlapping sites of lung, unspecified laterality (CMS/HCC) |
| C34.81 | Small cell lung cancer, overlapping sites of right lung (CMS/HCC) |
| C34.90 | Non-small cell lung cancer, unspecified laterality (CMS/HCC) |
| C34.91 | Non-small cell cancer of right lung (CMS/HCC) |
| C34.92 | Non-small cell cancer of left lung (CMS/HCC) |
| C38.3 | Primary mediastinal seminoma (CMS/HCC) |
| C38.4 | Malignant neoplasm of pleura, unspecified site |
| C40.12 | Cancer of fifth metacarpal bone of left hand (CMS/HCC) |
| C40.22 | Malignant neoplasm of long bone of left lower extremity (CMS/HCC) |
| C40.90 | Malignant neoplasm of unspecified bones and articular cartilage of unspecified limb (CMS/HCC) |
| C41.2 | Chordoma (CMS/HCC) |
| C41.4 | High grade osteosarcoma of pelvis (CMS/HCC) |
| C41.9 | Ewing's sarcoma of bone (CMS/HCC) |
| C48.2 | Primary peritoneal carcinomatosis (CMS/HCC) |
| C49.21 | Liposarcoma of thigh, right (CMS/HCC) |
| C49.22 | Liposarcoma of lower extremity, left (CMS/HCC) |
| C49.4 | Gastric liposarcoma (CMS/HCC) |
| C49.9 | Liposarcoma (CMS/HCC) |
| C49.A0 | Malignant gastrointestinal stromal tumor, unspecified site (CMS/HCC) |
| C49.A3 | Gastrointestinal stromal tumor (GIST) of duodenum (CMS/HCC) |
| C4A.62 | Merkel cell carcinoma of upper extremity, left (CMS/HCC) |
| C50.011 | Malignant neoplasm of nipple of right breast in female, unspecified estrogen receptor status (CMS/HCC) |
| C50.011, C50.012 | Bilateral malignant neoplasm involving both nipple and areola in female, unspecified estrogen receptor status (CMS/HCC) |
| C50.011, C50.012, Z17.0 | Malignant neoplasm of nipple of both breasts in female, estrogen receptor positive (CMS/HCC) |
| C50.011, Z17.0 | Malignant neoplasm of nipple of right breast in female, estrogen receptor positive (CMS/HCC) |
| C50.012 | Malignant neoplasm of areola of left breast in female, unspecified estrogen receptor status (CMS/HCC) |
| C50.022, Z17.0 | Malignant neoplasm involving both nipple and areola of left breast in male, estrogen receptor positive (CMS/HCC) |
| C50.111 | Malignant neoplasm of central portion of right female breast, unspecified estrogen receptor status (CMS/HCC) |
| C50.111, Z17.0 | Malignant neoplasm of central portion of right breast in female, estrogen receptor positive (CMS/HCC) |
| C50.112 | Malignant neoplasm of central portion of left female breast, unspecified estrogen receptor status (CMS/HCC) |
| C50.112, Z17.0 | Malignant neoplasm of central portion of left breast in female, estrogen receptor positive (CMS/HCC) |
| C50.122, Z17.0 | Malignant neoplasm of central portion of left breast in male, estrogen receptor positive (CMS/HCC) |
| C50.129 | Malignant neoplasm of central portion of male breast, unspecified laterality |
| C50.211 | Breast cancer of upper-inner quadrant of right female breast (CMS/HCC) |
| C50.211, Z17.0 | Malignant neoplasm of upper-inner quadrant of right breast in female, estrogen receptor positive (CMS/HCC) |
| C50.212 | Malignant neoplasm of upper-inner quadrant of left female breast, unspecified estrogen receptor status (CMS/HCC) |
| C50.212, Z17.0 | Carcinoma of upper-inner quadrant of left breast in female, estrogen receptor positive (CMS/HCC) |
| C50.212, Z17.1 | Malignant neoplasm of upper-inner quadrant of left breast in female, estrogen receptor negative (CMS/HCC) |
| C50.219 | Carcinoma of upper-inner quadrant of breast (CMS/HCC) |
| C50.311, Z17.0 | Malignant neoplasm of lower-inner quadrant of right breast of female, estrogen receptor positive (CMS/HCC) |
| C50.312, Z17.0 | Malignant neoplasm of lower-inner quadrant of left breast in female, estrogen receptor positive (CMS/HCC) |
| C50.312, Z17.1 | Malignant neoplasm of lower-inner quadrant of left breast in female, estrogen receptor negative (CMS/HCC) |
| C50.411 | Breast cancer of upper-outer quadrant of right female breast (CMS/HCC) |
| C50.411, C50.412 | Bilateral malignant neoplasm of upper outer quadrant of breast in female, unspecified estrogen receptor status (CMS/HCC) |
| C50.411, Z17.0 | Malignant neoplasm of upper-outer quadrant of right breast in female, estrogen receptor positive (CMS/HCC) |
| C50.411, Z17.1 | Malignant neoplasm of upper-outer quadrant of right breast in female, estrogen receptor negative (CMS/HCC) |
| C50.412 | Malignant neoplasm of upper-outer quadrant of left female breast (CMS/HCC) |
| C50.412, Z17.0 | Malignant neoplasm of upper-outer quadrant of left breast in female, estrogen receptor positive (CMS/HCC) |
| C50.412, Z17.1 | Malignant neoplasm of upper-outer quadrant of left breast in female, estrogen receptor negative (CMS/HCC) |
| C50.419, Z17.0 | Malignant neoplasm of upper-outer quadrant of breast in female, estrogen receptor positive, unspecified laterality (CMS/HCC) |
| C50.422, Z17.0 | Malignant neoplasm of upper-outer quadrant of left breast in male, estrogen receptor positive (CMS/HCC) |
| C50.511 | Malignant neoplasm of lower-outer quadrant of right female breast, unspecified estrogen receptor status (CMS/HCC) |
| C50.511, Z17.0 | Malignant neoplasm of lower-outer quadrant of right breast of female, estrogen receptor positive (CMS/HCC) |
| C50.511, Z17.1 | Malignant neoplasm of lower-outer quadrant of right breast of female, estrogen receptor negative (CMS/HCC) |
| C50.512 | Breast cancer of lower-outer quadrant of left female breast (CMS/HCC) |
| C50.512, Z17.0 | Malignant neoplasm of lower-outer quadrant of left breast of female, estrogen receptor positive (CMS/HCC) |
| C50.611 | Malignant neoplasm of axillary tail of right female breast, unspecified estrogen receptor status (CMS/HCC) |
| C50.612 | Malignant neoplasm of axillary tail of left female breast (CMS/HCC) |
| C50.612, Z17.0 | Malignant neoplasm of axillary tail of left breast in female, estrogen receptor positive (CMS/HCC) |
| C50.811 | Cancer of overlapping sites of right breast (CMS/HCC) |
| C50.811, C50.812, Z17.1 | Malignant neoplasm of overlapping sites of both breasts in female, estrogen receptor negative (CMS/HCC) |
| C50.811, Z17.0 | Malignant neoplasm of overlapping sites of right breast in female, estrogen receptor positive (CMS/HCC) |
| C50.811, Z17.1 | Malignant neoplasm of overlapping sites of right breast in female, estrogen receptor negative (CMS/HCC) |
| C50.812 | Malignant neoplasm of overlapping sites of left female breast, unspecified estrogen receptor status (CMS/HCC) |
| C50.812, Z17.0 | Malignant neoplasm of overlapping sites of left breast in female, estrogen receptor positive (CMS/HCC) |
| C50.812, Z17.1 | Malignant neoplasm of overlapping sites of left breast in female, estrogen receptor negative (CMS/HCC) |
| C50.819 | Overlapping malignant neoplasm of female breast, unspecified estrogen receptor status, unspecified laterality (CMS/HCC) |
| C50.911 | Infiltrating ductal carcinoma of right breast (CMS/HCC) |
| C50.911, C50.912 | Bilateral malignant neoplasm of breast in female, unspecified estrogen receptor status, unspecified site of breast (CMS/HCC) |
| C50.911, C77.3 | Breast cancer metastasized to axillary lymph node, right (CMS/HCC) |
| C50.911, Z17.0 | Malignant neoplasm of right breast in female, estrogen receptor positive, unspecified site of breast (CMS/HCC) |
| C50.911, Z17.0, C50.912 | Bilateral malignant neoplasm of breast in female, estrogen receptor positive, unspecified site of breast (CMS/HCC) |
| C50.911, Z17.1 | Malignant neoplasm of right breast in female, estrogen receptor negative, unspecified site of breast (CMS/HCC) |
| C50.912 | Malignant neoplasm of left female breast, unspecified site of breast |
| C50.912, C77.3 | Breast cancer metastasized to axillary lymph node, left (CMS/HCC) |
| C50.912, Z17.0 | Malignant neoplasm of left breast in female, estrogen receptor positive, unspecified site of breast (CMS/HCC) |
| C50.912, Z17.1 | Malignant neoplasm of left breast in female, estrogen receptor negative, unspecified site of breast (CMS/HCC) |
| C50.919 | Malignant neoplasm of breast (female) (CMS/HCC) |
| C50.919, C79.49 | Carcinoma of breast metastatic to central nervous system, unspecified laterality (CMS/HCC) |
| C50.919, C79.51 | Carcinoma of breast metastatic to bone, unspecified laterality (CMS/HCC) |
| C50.919, Z17.0 | Malignant neoplasm of breast in female, estrogen receptor positive, unspecified laterality, unspecified site of breast (CMS/HCC) |
| C50.929 | Breast cancer, male (CMS/HCC) |
| C51.9 | Vulvar cancer (CMS/HCC) |
| C52 | Vaginal cancer (CMS/HCC) |
| C53.0 | Malignant neoplasm of endocervix (CMS/HCC) |
| C53.8 | Malignant neoplasm of overlapping sites of cervix (CMS/HCC) |
| C53.9 | Cervical carcinoma (CMS/HCC) |
| C54.1 | Endometrial cancer (CMS/HCC) |
| C54.8 | Malignant neoplasm of overlapping sites of body of uterus (CMS/HCC) |
| C54.9 | Malignant neoplasm of body of uterus, unspecified site (CMS/HCC) |
| C55 | Leiomyosarcoma of uterus (CMS/HCC) |
| C56.1 | Ovarian CA, right (CMS/HCC) |
| C56.1, C56.2 | Malignant neoplasm of both ovaries (CMS/HCC) |
| C56.2 | Endometrioid adenocarcinoma of ovary, left (CMS/HCC) |
| C56.9 | Malignant neoplasm of ovary, unspecified laterality (CMS/HCC) |
| C57.9 | Gynecologic malignancy (CMS/HCC) |
| C60.9 | Penile cancer (CMS/HCC) |
| C61 | Prostate cancer (CMS/HCC) |
| C61, C77.2 | Prostate cancer metastatic to intraabdominal lymph node (CMS/HCC) |
| C61, C77.5 | Prostate cancer metastatic to intrapelvic lymph node (CMS/HCC) |
| C61, C79.51 | Malignant neoplasm of prostate metastatic to bone (CMS/HCC) |
| C62.01 | Malignant neoplasm of undescended right testis (CMS/HCC) |
| C62.10 | Malignant neoplasm of descended testis, unspecified laterality (CMS/HCC) |
| C62.11 | Seminoma of descended right testis (CMS/HCC) |
| C62.12 | Malignant neoplasm of descended left testis (CMS/HCC) |
| C62.90 | Seminoma, unspecified laterality (CMS/HCC) |
| C62.91 | Malignant neoplasm of right testis, unspecified whether descended or undescended (CMS/HCC) |
| C62.92 | Malignant neoplasm of left testis, unspecified whether descended or undescended (CMS/HCC) |
| C64.1 | Renal cell carcinoma of right kidney (CMS/HCC) |
| C64.2 | Renal cell carcinoma of left kidney (CMS/HCC) |
| C64.9 | Renal cell carcinoma, unspecified laterality (CMS/HCC) |
| C65.1 | Cancer of renal calyces, right (CMS/HCC) |
| C65.1, C66.1 | Cancer of right renal pelvis and ureter (CMS/HCC) |
| C65.9 | Malignant neoplasm of renal pelvis, unspecified laterality (CMS/HCC) |
| C66.1 | Ureteral cancer, right (CMS/HCC) |
| C66.9 | Malignant neoplasm of ureter, unspecified laterality (CMS/HCC) |
| C67.0 | Malignant neoplasm of trigone of urinary bladder (CMS/HCC) |
| C67.1 | Malignant neoplasm of dome of urinary bladder (CMS/HCC) |
| C67.2 | Malignant neoplasm of lateral wall of urinary bladder (CMS/HCC) |
| C67.3 | Malignant neoplasm of anterior wall of urinary bladder (CMS/HCC) |
| C67.4 | Malignant neoplasm of posterior wall of urinary bladder (CMS/HCC) |
| C67.8 | Malignant neoplasm of overlapping sites of bladder (CMS/HCC) |
| C67.9 | Malignant neoplasm of urinary bladder, unspecified site (CMS/HCC) |
| C68.9 | Urothelial cancer (CMS/HCC) |
| C69.01 | Malignant neoplasm of conjunctiva, right (CMS/HCC) |
| C69.11 | Malignant neoplasm of right cornea (CMS/HCC) |
| C69.20 | Retinoblastoma, unspecified laterality (CMS/HCC) |
| C69.21 | Retinoblastoma of right eye (CMS/HCC) |
| C69.21, C69.22 | Bilateral retinoblastoma (CMS/HCC) |
| C69.22 | Retinoblastoma of left eye (CMS/HCC) |
| C69.30 | Choroid carcinoma, unspecified laterality (CMS/HCC) |
| C69.90 | Ocular melanoma, unspecified laterality (CMS/HCC) |
| C69.92 | Malignant neoplasm of left eye (CMS/HCC) |
| C71.0 | Cerebral astrocytoma (CMS/HCC) |
| C71.1 | Malignant neoplasm of frontal lobe (CMS/HCC) |
| C71.3 | Oligoastrocytoma of parietal lobe (CMS/HCC) |
| C71.6 | Medulloblastoma (CMS/HCC) |
| C71.8 | Malignant neoplasm of overlapping sites of brain (CMS/HCC) |
| C71.9 | Astrocytoma brain tumor (CMS/HCC) |
| C71.9, F02.80 | Dementia due to primary malignant neoplasm of brain (CMS/HCC) |
| C72.0 | Ependymoma of spinal cord (CMS/HCC) |
| C72.30 | Optic nerve glioma (CMS/HCC) |
| C73 | Thyroid cancer (CMS/HCC) |
| C74.10 | Pheochromoblastoma, unspecified laterality (CMS/HCC) |
| C74.90 | Neuroblastoma (CMS/HCC) |
| C75.0 | Malignant neoplasm of parathyroid gland (CMS/HCC) |
| C76.0 | Squamous cell carcinoma of head and neck (CMS/HCC) |
| C76.3 | Cancer of perineum (CMS/HCC) |
| C77.0 | Metastatic cancer to cervical lymph nodes (CMS/HCC) |
| C77.2 | Metastatic cancer to intra-abdominal lymph nodes (CMS/HCC) |
| C77.3 | Malignant neoplasm metastatic to lymph node of axilla (CMS/HCC) |
| C77.9 | Metastatic squamous cell carcinoma to lymph node (CMS/HCC) |
| C77.9, C64.9 | Metastatic renal cell carcinoma to lymph node (CMS/HCC) |
| C77.9, C80.1 | Secondary squamous cell carcinoma of lymph node with unknown primary site (CMS/HCC) |
| C78.00 | Malignant neoplasm metastatic to lung, unspecified laterality (CMS/HCC) |
| C78.01, C80.1 | Metastatic adenocarcinoma involving right lung with unknown primary site (CMS/HCC) |
| C78.1, C80.1 | Metastatic squamous cell carcinoma involving mediastinum with unknown primary site (CMS/HCC) |
| C78.5, C80.1 | Malignant neoplasm metastatic to colon with unknown primary site (CMS/HCC) |
| C78.6, C80.1 | Peritoneal carcinomatosis (CMS/HCC) |
| C78.7 | Metastatic carcinoma to liver (CMS/HCC) |
| C78.7, C80.1 | Metastasis to liver with unknown primary site (CMS/HCC) |
| C79.10 | Metastatic urothelial carcinoma (CMS/HCC) |
| C79.31 | Brain metastases (CMS/HCC) |
| C79.49 | Metastasis to spinal cord (CMS/HCC) |
| C79.51 | Bone metastasis (CMS/HCC) |
| C79.51, C80.1 | Metastatic adenocarcinoma involving skeletal bone with unknown primary site (CMS/HCC) |
| C79.52 | Metastatic adenocarcinoma to bone marrow (CMS/HCC) |
| C79.70, C22.8 | Secondary hepatocellular carcinoma of adrenal gland, unspecified laterality (CMS/HCC) |
| C79.81 | Metastatic malignant neoplasm to breast (CMS/HCC) |
| C79.89 | Secondary malignancy of shoulder (CMS/HCC) |
| C79.89, C80.1 | Squamous cell carcinoma metastatic to head and neck with unknown primary site (CMS/HCC) |
| C79.9 | Multiple lesions of metastatic malignancy (CMS/HCC) |
| C79.9, C25.9 | Metastasis from pancreatic cancer (CMS/HCC) |
| C7A.090 | Malignant carcinoid tumor of lung (CMS/HCC) |
| C7A.098 | Malignant carcinoid tumors of other sites (CMS/HCC) |
| C7A.8 | Neuro-endocrine carcinoma (CMS/HCC) |
| C7B.8 | Metastatic malignant neuroendocrine tumor to lymph node (CMS/HCC) |
| C80.0 | Carcinomatosis (CMS/HCC) |
| C80.0, C80.1 | Disseminated malignancy of unknown primary (CMS/HCC) |
| C80.1 | Adenocarcinoma of unknown origin (CMS/HCC) |
| C80.1, M36.1 | Arthropathy associated with cancer (CMS/HCC) |
| C81.01 | Nodular lymphocyte predominant Hodgkin lymphoma of lymph nodes of neck (CMS/HCC) |
| C81.10 | Nodular sclerosing Hodgkin's lymphoma, unspecified body region (CMS/HCC) |
| C81.40 | Lymphocyte-rich Hodgkin lymphoma, unspecified body region (CMS/HCC) |
| C81.48 | Lymphocyte-rich classic Hodgkin lymphoma lymph nodes multiple sites (CMS/HCC) |
| C81.74 | Other classical Hodgkin lymphoma of lymph nodes of axilla (CMS/HCC) |
| C81.90 | Hodgkin lymphoma, unspecified Hodgkin lymphoma type, unspecified body region (CMS/HCC) |
| C81.91 | Hodgkin lymphoma of lymph nodes of neck, unspecified Hodgkin lymphoma type (CMS/HCC) |
| C81.94 | Hodgkin lymphoma of lymph nodes of axilla, unspecified Hodgkin lymphoma type (CMS/HCC) |
| C81.98 | Hodgkin lymphoma of lymph nodes of multiple regions, unspecified Hodgkin lymphoma type (CMS/HCC) |
| C81.99 | Hodgkin lymphoma of extranodal or solid organ site (CMS/HCC) |
| C82.00 | Follicular lymphoma grade I, unspecified body region (CMS/HCC) |
| C82.03 | Follicular lymphoma grade I of intra-abdominal lymph nodes (CMS/HCC) |
| C82.10 | Follicular lymphoma grade II, unspecified body region (CMS/HCC) |
| C82.12 | Follicular lymphoma grade II of intrathoracic lymph nodes (CMS/HCC) |
| C82.30 | Follicular lymphoma grade IIIa, unspecified body region (CMS/HCC) |
| C82.50 | Diffuse follicle center lymphoma, unspecified body region (CMS/HCC) |
| C82.80 | Follicular low grade B-cell lymphoma (CMS/HCC) |
| C82.90 | Follicular non-Hodgkin's lymphoma (CMS/HCC) |
| C82.98 | Follicular lymphoma of lymph nodes of multiple regions, unspecified follicular lymphoma type (CMS/HCC) |
| C82.99 | Follicular lymphoma of extranodal site excluding spleen and other solid organs, unspecified grade (CMS/HCC) |
| C83.00 | Small B-cell lymphoma, unspecified body region (CMS/HCC) |
| C83.01 | Small B-cell lymphoma of lymph nodes of head (CMS/HCC) |
| C83.10 | Mantle cell lymphoma, unspecified body region (CMS/HCC) |
| C83.30 | Diffuse large B-cell lymphoma, unspecified body region (CMS/HCC) |
| C83.31 | Diffuse large B-cell lymphoma of lymph nodes of head (CMS/HCC) |
| C83.33 | Diffuse large B-cell lymphoma of intra-abdominal lymph nodes (CMS/HCC) |
| C83.34 | Diffuse large B-cell lymphoma of lymph nodes of axilla (CMS/HCC) |
| C83.35 | Diffuse large B-cell lymphoma of lymph nodes of inguinal region (CMS/HCC) |
| C83.37 | Diffuse large B-cell lymphoma of spleen (CMS/HCC) |
| C83.38 | Diffuse large B-cell lymphoma of lymph nodes of multiple regions (CMS/HCC) |
| C83.39 | Diffuse large B-cell lymphoma of solid organ excluding spleen (CMS/HCC) |
| C83.73 | Burkitt lymphoma of intra-abdominal lymph nodes (CMS/HCC) |
| C84.00 | Mycosis fungoides, unspecified body region (CMS/HCC) |
| C84.08 | Mycosis fungoides involving lymph nodes of multiple regions (CMS/HCC) |
| C84.40 | Angioimmunoblastic lymphoma (CMS/HCC) |
| C84.47 | Peripheral T cell lymphoma, splenic (CMS/HCC) |
| C84.49 | Peripheral T cell lymphoma of extranodal and solid organ sites (CMS/HCC) |
| C84.A0 | Pleomorphic small or medium-sized cell cutaneous T-cell lymphoma (CMS/HCC) |
| C84.A8 | Cutaneous T-cell lymphoma involving lymph nodes of multiple regions (CMS/HCC) |
| C85.10 | B-cell lymphoma, unspecified B-cell lymphoma type, unspecified body region (CMS/HCC) |
| C85.11 | B-cell lymphoma of lymph nodes of neck, unspecified B-cell lymphoma type (CMS/HCC) |
| C85.80 | Malignant lymphoma, undifferentiated cell, non-Burkitt's (CMS/HCC) |
| C85.81 | Other specified type of non-Hodgkin lymphoma of neck (CMS/HCC) |
| C85.89 | CNS lymphoma (CMS/HCC) |
| C85.90 | Lymphoma, unspecified body region, unspecified lymphoma type (CMS/HCC) |
| C85.90, G63 | Motor neuropathy with lymphoma (CMS/HCC) |
| C85.91 | Non-Hodgkin lymphoma of lymph nodes of neck, unspecified non-Hodgkin lymphoma type (CMS/HCC) |
| C85.93 | Lymphoma of intra-abdominal lymph nodes, unspecified lymphoma type (CMS/HCC) |
| C85.94 | Lymphoma of lymph nodes of axilla, unspecified lymphoma type (CMS/HCC) |
| C85.95 | Non-Hodgkin lymphoma of lymph nodes of inguinal region, unspecified non-Hodgkin lymphoma type (CMS/HCC) |
| C85.97 | Non-Hodgkin's lymphoma of spleen, unspecified non-Hodgkin lymphoma type (CMS/HCC) |
| C85.98 | Lymphoma of lymph nodes of multiple regions, unspecified lymphoma type (CMS/HCC) |
| C85.99 | Non-Hodgkin lymphoma of extranodal site excluding spleen and other solid organs, unspecified non-Hodgkin lymphoma type (CMS/HCC) |
| C86.6 | Lymphomatoid papulosis (CMS/HCC) |
| C88.0 | Waldenstrom macroglobulinemia (CMS/HCC) |
| C88.4 | MALT lymphoma (CMS/HCC) |
| C90.00 | Multiple myeloma not having achieved remission (CMS/HCC) |
| C90.00, C88.9 | Multiple myeloma and immunoproliferative neoplasms (CMS/HCC) |
| C90.01 | Multiple myeloma in remission (CMS/HCC) |
| C90.02 | Multiple myeloma in relapse (CMS/HCC) |
| C90.20 | Extramedullary plasmacytoma of paranasal sinus (CMS/HCC) |
| C91.00 | ALL (acute lymphoblastic leukemia of infant) (CMS/HCC) |
| C91.01 | ALL (acute lymphoid leukemia) in remission (CMS/HCC) |
| C91.10 | CLL (chronic lymphocytic leukemia) (CMS/HCC) |
| C91.40 | Hairy cell leukemia not having achieved remission (CMS/HCC) |
| C91.41 | Hairy cell leukemia, in remission (CMS/HCC) |
| C91.60 | Prolymphocytic leukemia of T-cell (CMS/HCC) |
| C91.91 | Lymphoid leukemia in remission, unspecified lymphoid leukemia type (CMS/HCC) |
| C91.Z0 | Large granular lymphocytic leukemia (CMS/HCC) |
| C92.00 | Acute myeloid leukemia not having achieved remission (CMS/HCC) |
| C92.01 | AML (acute myeloid leukemia) in remission (CMS/HCC) |
| C92.10 | CML (chronic myelocytic leukemia) (CMS/HCC) |
| C92.30 | Myeloid sarcoma, not having achieved remission (CMS/HCC) |
| C92.40 | Acute promyelocytic leukemia not having achieved remission (CMS/HCC) |
| C92.41 | Acute promyelocytic leukemia in remission (CMS/HCC) |
| C93.10 | Chronic myelomonocytic leukemia not having achieved remission (CMS/HCC) |
| C93.11 | Chronic myelomonocytic leukemia, in remission (CMS/HCC) |
| C95.00 | Acute leukemia not having achieved remission (CMS/HCC) |
| C95.01 | Acute leukemia in remission (CMS/HCC) |
| C95.90 | Leukemia not having achieved remission (CMS/HCC) |
| C95.91 | Leukemia in remission, unspecified leukemia type (CMS/HCC) |
| C95.92 | Leukemia in relapse, unspecified leukemia type (CMS/HCC) |
| C96.6 | Langerhan's cell histiocytosis (CMS/HCC) |
| D01.3 | Severe anal dysplasia |
| D02.20 | Carcinoma in situ of bronchus or lung |
| D03.61 | Melanoma in situ of right shoulder (CMS/HCC) |
| D03.9 | Melanoma in situ, unspecified site (CMS/HCC) |
| D04.30 | Squamous cell carcinoma in situ (SCCIS) of skin of face |
| D04.9 | Basal cell carcinoma in situ of skin |
| D05.00 | Lobular carcinoma in situ (LCIS) of breast, unspecified laterality |
| D05.01 | Lobular carcinoma in situ (LCIS) of right breast |
| D05.02 | Lobular carcinoma in situ of left breast |
| D05.10 | Ductal carcinoma in situ (DCIS) of breast, unspecified laterality |
| D05.11 | Ductal carcinoma in situ (DCIS) of right breast |
| D05.11, D05.12 | Bilateral ductal carcinoma in situ of breasts |
| D05.12 | DCIS (ductal carcinoma in situ) of breast, left |
| D05.90 | Breast cancer in situ |
| D05.91 | Carcinoma in situ of right breast |
| D05.92 | Carcinoma in situ of left breast |
| D06.0 | Carcinoma in situ of endocervix |
| D06.1 | Carcinoma in situ of exocervix |
| D06.7 | Carcinoma in situ of other part of cervix |
| D06.9 | CIN III (cervical intraepithelial neoplasia grade III) with severe dysplasia |
| D07.1 | Vulvar intraepithelial neoplasia (VIN) grade 3 |
| D07.2 | Severe vaginal dysplasia |
| D07.30 | Adenocarcinoma in situ of female genital organ |
| D09.0 | Bladder CA in situ |
| D09.9 | Squamous cell carcinoma in situ |
| D37.030 | Neoplasm of uncertain behavior of parotid gland |
| D37.05 | Neoplasm of uncertain behavior of oropharynx |
| D37.09 | Neoplasm of uncertain behavior of upper gingiva |
| D37.3 | Neoplasm of uncertain behavior of appendix |
| D37.4 | Neoplasm of uncertain behavior of ascending colon |
| D37.6 | Neoplasm of uncertain behavior of liver and biliary passages |
| D37.8 | Neoplasm of uncertain behavior of other specified digestive organs |
| D37.9 | Gastrinoma |
| D38.0 | Neoplasm of uncertain behavior of larynx |
| D39.0 | Diffuse leiomyomatosis of uterus |
| D39.10 | Ovarian neoplasm with low malignant potential |
| D39.11 | Ovarian stromal tumor, right |
| D39.12 | Granulosa cell tumor of left ovary |
| D39.2 | Placental site nodule |
| D39.8 | Neoplasm of uncertain behavior of clitoris |
| D3A.00 | Carcinoid tumor |
| D3A.012 | Carcinoid tumor of ileum |
| D3A.019 | Benign neuroendocrine tumor of small intestine |
| D3A.026 | Rectal carcinoid tumor |
| D3A.090 | Carcinoid tumor determined by biopsy of lung |
| D3A.092 | Carcinoid tumor determined by biopsy of stomach |
| D3A.098 | Carcinoid tumor of abdomen |
| D3A.8 | Neuroendocrine neoplasm of gastrointestinal tract |
| D41.02 | Hemangiopericytoma of kidney, left |
| D41.4 | Neoplasm of uncertain behavior of anterior wall of urinary bladder |
| D43.2 | Neoplasm of uncertain behavior of brain (CMS/HCC) |
| D44.0 | Neoplasm of uncertain behavior of thyroid gland |
| D44.10 | Mass of uncertain behavior of adrenal gland |
| D44.4 | Adamantinous craniopharyngioma (CMS/HCC) |
| D44.6 | Carotid body tumor (CMS/HCC) |
| D44.7 | Glomus tympanicum tumor (CMS/HCC) |
| D47.9 | Lymphoproliferative disease (CMS/HCC) |
| D47.Z2 | Castleman disease (CMS/HCC) |
| D47.Z9 | Lymphoproliferative disorder, low grade B cell (CMS/HCC) |
| D48.0 | Neoplasm of bone of left foot |
| D48.1 | Giant cell tumor of tendon sheath |
| D48.4 | Neoplasm of uncertain behavior of mesentery |
| D48.60 | Phyllodes tumor of breast |
| D48.61 | Cystosarcoma of breast, right |
| D48.62 | Neoplasm of uncertain behavior of left female breast |
| D48.7 | Neoplasm of uncertain behavior of nose |
| D48.9 | Neoplasm of uncertain behavior |
| D49.0 | Pancreas neoplasm |
| D49.1 | Lung tumor |
| D49.2 | Skin growth |
| D49.3 | Neoplasm of breast |
| D49.4 | Neoplasm of bladder |
| D49.519 | Kidney tumor |
| D49.59 | Neoplasm of prostate |
| D49.6 | Brain tumor (CMS/HCC) |
| D49.7 | Thyroid neoplasm |
| D49.81 | Neoplasm of unspecified behavior of retina and choroid |
| D49.89 | Lacrimal gland tumor |

| **Supplemental Table 2.** Entire list of cancers in this study, comparing cases to controls. | | | | |
| --- | --- | --- | --- | --- |
| **Cancer type** | **Total (3699)** | **ED** | **Non-ED** | **p value** |
| adrenal | 1 (0.03) | 1 (0.08%) | 0 | 0.159 |
| anal | 3 (.08%) | 1 (0.08) | 2 (0.08) | 0.9952 |
| bladder | 117 (3.16) | 36 (2.91%) | 81 (3.29%) | 0.5255 |
| bone | 30 (0.81) | 9 (0.73%) | 21 (0.85%) | 0.684 |
| brain | 121 (3.27) | 25 (2.02%) | 96 (3.90%) | 0.0025 |
| breast | 572 (15.46%) | 126 (10.17%) | 449 (18.18%) | <.0001 |
| cervical | 235 (6.33%) | 63 (5.08%) | 171 (6.95%) | 0.028 |
| CRC | 209 (5.65%) | 85 (6.86%) | 124 (5.04%) | 0.0237 |
| esophagus | 22 (0.59%) | 8 (0.65%) | 14 (0.57%) | 0.775 |
| HEENT | 359 (9.91%) | 109 (8.80%) | 250 (10.16%) | 0.1856 |
| kidney | 123 (3.33%) | 53 (4.28%) | 70 (2.85%) | 0.0218 |
| leukemia | 74 (2.0%) | 20 (1.61%) | 54 (2.20%) | 0.2337 |
| liposarcoma | 20 (0.54%) | 9 (0.73%) | 11 (0.45%) | 0.2744 |
| liver | 103 (2.78%) | 54 (4.28%) | 50 (2.03%) | <.0001 |
| lung | 282 (7.62%) | 145 (11.70%) | 137 (5.57%) | <.0001 |
| lymph nodes | 21 (0.57%) | 8 (0.65%) | 13 (0.53% | 0.654 |
| lymphoma | 142 (3.8%) | 55 (1.49%) | 87 (2.4%) | 0.1776 |
| merkel cell | 1 (0.04%) | 0 | 1 (0.03%) |  |
| metastatic unk primary | 180 (4.87%) | 57 (4.60%) | 123 (5.0%) | 0.594 |
| myeloma | 37 (1.0%) | 10 (0.81%) | 27 (1.10%) | 0.4021 |
| neuroendocrine | 27 (.73%) | 8 (0.65%) | 27 (1.10%) | 0.6693 |
| ocular | 13 (0.35%) | 1 (0.08%) | 12 (0.49%) | 0.0483 |
| oral | 79 (2.14%) | 25 (2.02%) | 54 (2.20%) | 0.7247 |
| ovarian | 38 (1.03%) | 12 (0.97%) | 26 (1.06%) | 0.8013 |
| pancreatic | 200 (5.41%) | 92 (7.43%) | 108 (4.39%) | 0.0001 |
| parathyroid | 2 (0.05%) | 1 (0.08%) | 1 (0.03%) | 0.6208 |
| penis | 3 (.08%) | 2 (0.16%) | 1 (0.03%) | 0.2233 |
| perineum | 1 (0.03) | 1 (0.08%) | 0 |  |
| peritoneal | 1 (0.03) | 0 | 1 (0.03%) |  |
| prostate | 312 (8.43%) | 98 (7.91%) | 214 (8.70%) | 0.4147 |
| small bowel | 26 | 8 (0.65%) | 18 (0.73%) | 0.7676 |
| stomach | 27 (0.73%) | 8 (0.65%) | 18 (0.73%) | 0.0044 |
| testicular | 19 (0.51%) | 7 (0.56%) | 12 (0.49%) | 0.7567 |
| thyroid | 165 (4.46%) | 42 (3.39%) | 123 (5.0%) | 0.0252 |
| unknown | 17 (0.46%) | 5 (0.40%) | 12 (0.49%) | 0.7207 |
| ureteral | 6 (0.16) | 3 (0.24%) | 3 (0.12%) | 0.3913 |
| uterus | 75 (2.03%) | 28 (2.26%) | 47 (1.91%) | 0.4768 |
| vagina | 6 (0.16) | 3 (0.24%) | 3 (0.12%) | 0.0418 |
| vulva | 31 (0.84%) | 14 (1.13%) | 17 (0.69%) | 0.167 |
